# Investigation on Sensing Performance of Highly Doped Sb/SnO_2_

**DOI:** 10.3390/s22031233

**Published:** 2022-02-06

**Authors:** Zhifu Feng, Andrea Gaiardo, Matteo Valt, Barbara Fabbri, Davide Casotti, Soufiane Krik, Lia Vanzetti, Michele Della Ciana, Simona Fioravanti, Stefano Caramori, Alberto Rota, Vincenzo Guidi

**Affiliations:** 1 MNF—Micro Nano Facility, Sensors and Devices Center, Bruno Kessler Foundation, Via Sommarive 18, 38123 Trento, Italy; gaiardo@fbk.eu (A.G.); mvalt@fbk.eu (M.V.); vanzetti@fbk.eu (L.V.); sfioravanti@fbk.eu (S.F.); 2 Department of Physics and Earth Sciences, University of Ferrara, Via Saragat 1, 44122 Ferrara, Italy; barbara.fabbri@unife.it (B.F.); dellaciana@bo.imm.cnr.it (M.D.C.); 3 CNR—Institute of Nanoscience, Centro S3, Via Campi 213/A, 41125 Modena, Italy; davide.casotti@unimore.it (D.C.); alberto.rota@unimore.it (A.R.); 4 Faculty of Science and Technology, Free University of Bozen-Bolzano, Piazza Università 1, 39100 Bozen, Italy; soufiane.krik@unibz.it; 5 Unit of Bologna, Institute for Microelectronics and Microsystems, National Research Council, Via Gobetti 101, 40129 Bologna, Italy; 6 Department of Chemical, Pharmaceutical and Agricultural Sciences, University of Ferrara, Via Luigi Borsari 46, 44121 Ferrara, Italy; cte@unife.it; 7 Department of Physics, Informatics and Mathematics, University of Modena and Reggio Emilia, Via Campi 213/A, 41125 Modena, Italy; 8 Interdepartmental Center for Applied Research and Services in the Advanced Mechanics and Motor Engineering Sector, University of Modena and Reggio Emilia, Via Vignolese 905/B, 41125 Modena, Italy

**Keywords:** tin dioxide, antimony doping, chemiresistive gas sensing, NO2 detection, humidity influence, nanostructured semiconductors

## Abstract

Tin dioxide (SnO_2_) is the most-used semiconductor for gas sensing applications. However, lack of selectivity and humidity influence limit its potential usage. Antimony (Sb) doped SnO_2_ showed unique electrical and chemical properties, since the introduction of Sb ions leads to the creation of a new shallow band level and of oxygen vacancies acting as donors in SnO_2_. Although low-doped SnO_2_:Sb demonstrated an improvement of the sensing performance compared to pure SnO_2_, there is a lack of investigation on this material. To fill this gap, we focused this work on the study of gas sensing properties of highly doped SnO_2_:Sb. Morphology, crystal structure and elemental composition were characterized, highlighting that Sb doping hinders SnO_2_ grain growth and decreases crystallinity slightly, while lattice parameters expand after the introduction of Sb ions into the SnO_2_ crystal. XRF and EDS confirmed the high purity of the SnO_2_:Sb powders, and XPS highlighted a higher Sb concentration compared to XRF and EDS results, due to a partial Sb segregation on superficial layers of Sb/SnO_2_. Then, the samples were exposed to different gases, highlighting a high selectivity to NO_2_ with a good sensitivity and a limited influence of humidity. Lastly, an interpretation of the sensing mechanism vs. NO_2_ was proposed.

## 1. Introduction

Air pollution is becoming increasingly severe as a result of industrial expansion and population growth. Numerous greenhouse gases and hazardous chemicals are released into the ambient air, including carbon oxides, nitrogen oxides, benzene, and ammonia, posing a harm to human health [1]. As a result, identifying hazardous and organic gases in environmental air is critical for natural protection and sustainable societal development. Optical gas sensors and chemiresistive gas sensors are often used for the detection of the above-mentioned target gases. Even though optical gas sensors gain the advantages of high selectivity and sensitivity, its system is fundamentally equipped with a light source and optical receiver, which makes its volume big and price high [2]. Semiconductor gas sensors are employed extensively due to their chemical stability, quick response, low-cost fabrication, and easy integration [3]. Semiconductor materials are classified into two types: n-type materials, such as SnO_2_, ZnO, WO_3_, Fe_2_O_3_, and TiO_2_, and p-type materials, such as Cr_2_O_3_, CuO, NiO, and Co_3_O_4_, whose resistance is greatly dependent on the surface states and easily altered by gas adsorption and desorption kinetics [4]. The fundamental sensing mechanism states that the conductivity of this type of sensor is controlled by the changing concentrations of unsaturated oxygen adsorption sites on the surface, which is determined by the balance of oxygen adsorption and surface reactivity between the oxygen adsorbate and target gases [5].

SnO_2_, with a wide band gap of about 3.6 eV at 300 K, is one of the most extensively used semiconductor materials for gas sensors due to its steady-state chemical and physical properties at various temperatures. However, drawbacks of SnO_2_-based gas sensors still need to be addressed, including high working temperature and poor selectivity [6,7]. Based on the sensing mechanism model [8], there are three reasonably effective strategies for increasing the sensitivity and selectivity of SnO_2_: nanostructure modification, heterojunction structure building, and noble metal elements doping. For instance, a slew of remarkable research works is devoted to synthesizing SnO_2_ with various nano-morphologies, such as nanowires [9], nanoflower [10,11], nanofibers [12], nano hollow [13], 2D flakes [14], nanosphere [15,16], 3D mesoporous [17], hollow sphere [18], and 3D microporous spheres [19,20]. However, it is extremely hard to find out the mechanisms of how the morphologies changing affects the sensing properties, and the nanostructures of the materials seem extremely difficult to be designed and predicted. A heterojunction structure can be created by compositing semiconductor materials with different Fermi levels, which can cause higher sensitivity and selectivity by adjusting the electron structures [21,22,23]. However, the heterojunction part is so sensitive to the composite materials semiconductor properties, content ratio, and structure; hence, the sensing results are not ideally repeatable. Moreover, doping with noble elements can change the position of energy levels in the conduction band of SnO_2_, such as by doping Pd [24], Ru [25], Al [26], Pt [27], Sb [28], Ce [29], Ag [30], and Ti [31], which is a more reliable and economical way to increase the selectivity and sensitivity compared to nanostructure modification and heterojunction structure building towards the gas sensing performance optimization [6].

Among these above-mentioned doping elements, Sb-doped SnO_2_ (ATO) acts as a transparent n-type semiconductor, which is widely employed as a transparent conducting thin film for solar cell, optoelectronic, and gas sensor material due to its quasi-metallic conductivity feature [11,32,33,34,35,36]. Sb ions could exist as Sb^3+^ or Sb^5+^ in SnO_2_ crystal structure depending on synthesis conditions (temperature, doping concentration, etc.) and on the temperature of the calcination treatment [37,38]. Therefore, Sb ions doping can significantly affect SnO_2_ electrical, electrochemical, and optical properties by affecting the conducting band bending degree and inserting a new shallow band level [39]. Indeed, the replacement of Sn^4+^ by Sb^3+^ introduces a shallow acceptor level close to the valence band, while the substitution by Sb^5+^ results in the formation of a shallow donor level close to the conduction band of SnO_2_ [40]. The ratio of Sb^5+^ and Sb^3+^ sites regulates the free carrier concentration on the doped material and defines the conductivity type [41]. Furthermore, the Sb doping usually leads to the creation of oxygen vacancies acting as donors in SnO_2_, generating extra electrons into the conduction band, and a single or double ionization can emerge to the vacancies [40,42].

Due to the unique semiconducting properties, ATO has been widely investigated as an active material in gas sensors. Gayatri Joshi et al. doped SnO_2_ by a maximum 2 at.% of antimony to improve the materials’ stability in the O_2_ environment, and they obtained the highest response for 1 at.% Sb-doped SnO_2_ thin-film sample [32]. Jae-Hun Kim et al. synthesized ATO nanowires by using ion implanter at different does of 2 × 10^13^, 2 × 10^14^ and 2 × 10^15^ ion/cm^2^, and described the sensing properties under different target gases NO_2_, O_2_, and SO_2_, where the lowest dose gas sensor exhibited the best performance in this case [28]. Qi Wei et al. designed a gas sensor based on a 5% mole ratio ATO as a sensing material to detect formaldehyde gas with different concentrations [11]. Jiang Ma et al. deposited ATO nanoribbons sensing material via thermal evaporation, which showed an extremely high response to H_2_S gas compared to other two toxic gases of NO_2_ and CO, and even at room temperature, this material exhibited relatively high sensitivity and good selectivity [35]. Kamalpreet Khun Khun et al. prepared the ATO powder with concentrations ranging from 0 to 6 mol% by means of nonaqueous sol-gel method, and studied the sensitivity to 50 ppm NH_3_, acetone and ethanol at room temperature (25 °C). The ATO-based gas sensors in their study showed a special selectivity to NH_3_ with a response value about 4316% for the samples having 4 mol% of Sb [43].

Based on other groups’ research works, it is well known that higher doping levels sometimes result in a sensing performance improvement of the active materials. M.C. Carotta et al. discovered that a higher titanium ratio (molar ratio 0.5 compared to 0.1 and 0) in SnO_2_ resulted in improved gas responses under dry and moisture CO at 550 °C and 600 °C [44]. S. Morandi et al. reported that a higher tin atom ratio (tin molar fraction 0.12 compared to 0 and 0.0018) in tungsten oxide exhibited higher response under NO_2_ at 250 °C [45]. A. Gaiardo et al. developed an iron oxide highly doped with samarium (SmFeO_3_) able to detect CO with a good repeatability, and finally used as sensing material in the SCENT prototype for the screening cancer [46,47]. To the best of our knowledge, highly Sb-doped SnO_2_ as a gas sensor material has not been investigated so far, and most doping levels are under 5 wt.% for gas sensing application.

Therefore, here, we have conducted an investigation to compare the sensing performance of pure nanostructured SnO_2_ (S1) and highly Sb-doped SnO_2_ by using two different concentrations of Sb (10 and 15 wt%), named as ATO1 and ATO2, respectively. The morphology, crystal structure, and stoichiometric information of the obtained samples were characterized by means of Scanning Electron Microscope (SEM), Energy Dispersive X-ray Spectroscopy (EDS), powder X-ray Diffraction (XRD), X-ray Fluorescence Spectrometer (XRF), and X-ray Photoelectron Spectroscopy (XPS). It revealed the influence of antimony doping on the morphology and crystallinity of SnO_2_, quantified the atoms ratio between tin and antimony by XRF and EDS, and proved the existences of Sn^4+^, Sb^3+^, and Sb^5+^ ions in the samples. Then, the films were drop-coated onto suitable substrates to obtain the final devices. The sensors were tested in presence of nine different common gases in order to study the selectivity after the material doping. In addition, the sensitivity and selectivity of fabricated devices towards NO_2_ at different temperatures in dry and wet air were investigated.

## 2. Materials and Methods

### 2.1. Samples Preparation

All three nanopowders were obtained by heat treatment from the commercial colloidal solution products (Alfa Aesar, Ward Hill, MA, USA), which are SnO_2_ solution (15% in water) and 10 wt% and 15 wt% Sb-doped SnO_2_ solution (50% in water) from Alfa Aesar (Ward Hill, MA, USA) as previously described in [48,49].

### 2.2. Measurement Techniques

Morphology and elemental composition were determined by the SEM and EDS, using a SEM Zeiss (Leo) Gemini 1530 (Jena, Germany) and Thermo Fisher Scientific Helios (Waltham, MA, USA) 5 CXe with energy of 5 keV and current of 0.1 nA. Crystal phase, grain size, and cell parameters were estimated by X-ray Diffraction (XRD) through a PANalytical X’Pert PRO (PANalytical B.V., Almelo, The Netherlands) instrument, equipped by X’Celerator detector based on Real Time Multi Strip Technology (RTMS) and optimized for Cu Kα1,2 radiation. The X-ray tube was set at 40 mA and 40 kV and the data were collected at room temperature. The X’Pert HighScore Plus version 2.0 program by PANalytical B.V., coupled with the Powder Diffraction File database (PDF), was utilized for phase identifications. The Profex v. 4.2.1 fitting program was applied to estimate the cell parameters and the average crystallite size by Rietveld and Le Bail fits, which were carried out with the fundamental parameters approach [50]. XRF analysis was conducted by HORIBA XGT-7200V (Horiba Italia Srl, Roma, Italy)with rhodium X-ray source. The work energy ranged from 15 to 30 keV, and the incident gun spot size changed between 10 and 100 μm to obtain reasonable results. XPS measurements were performed using a Scienta ESCA 200 instrument (Scientaomicron, Uppsala, Sweden) equipped with a hemispherical analyzer and a monochromatic Al Kα (1486.6 eV) X-ray source. The emission angle between the analyzer axis and the normal to the sample surface was 0°, corresponding to a sampling depth of approximately 10 nm. For each sample, O 1s, Sb 3d, Sn 3d, and C 1s core levels were recorded at a pass energy of 150 eV. The XPS spectra binding energy scale was calibrated by referencing the C 1s hydrocarbon peak at 284.5 eV. The quantification, reported as the relative elemental percentage, was made using the integrated area of the core levels, after Shirley background subtraction, and using atomic sensitivity factors. XPS data were analyzed using the software described in Speranza and Canteri [51].

For XPS and XRF measurements, the powders were placed freely on double-sided carbon tape that was adhered to the aluminum sample holder; all samples were analyzed at room temperature. The optical properties of the different samples were investigated by using a JASCO V-670 double beam spectrophotometer (JASCO International Co., Ltd., Tokyo, Japan). The instrument is equipped with both a deuterium lamp (190–350 nm) and a halogen one (330–2700 nm). The measurements were carried out in the wavelength range 250–1200 nm, and the wavelength interval used was 1 nm. For the characterization, the powders were dispersed in water. The S1, ATO1, and ATO2 band gaps were calculated by using Tauc plot method.

### 2.3. Sensor Preparation

Silicon microheaters were used to keep a steady operating temperature up to 500 °C and mechanical support for sensing materials. The sensing materials were drop-coated onto the top of the microheater. The microheater is composed of 120/10 nm thick Pt/Ti interdigitated electrodes and heater, deposited by electron beam evaporation on a 900 nm ONO stack layer (SiO_2_/Si_3_N_4_/SiO_2_), which was formed by thermal growth and low-pressure chemical vapor deposition processes above 300 μm thick silicon wafer with <100> crystal orientation and resistivity of 10–20 Ω∙cm [52,53].

**Figure 1:**
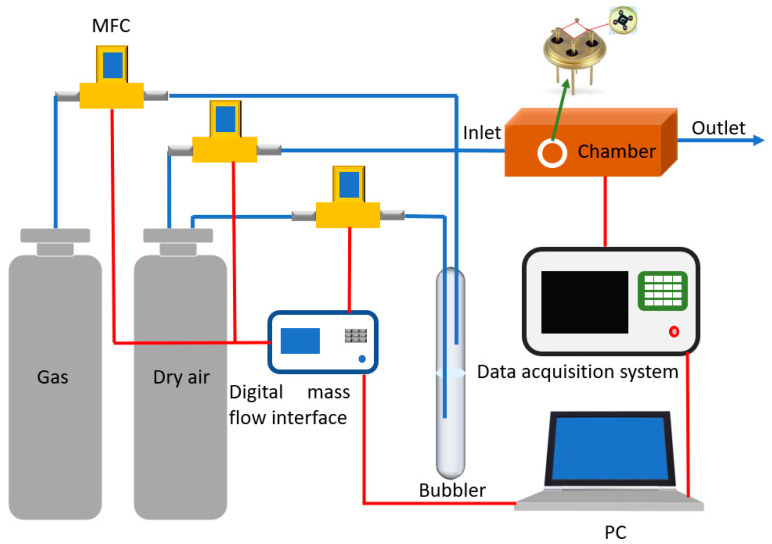
Scheme of the apparatus of the gas sensor characterization system.

To make the sensing films, the nanopowders were blended with suitable organics in order to prepare the screen-printable pastes, specifically mixed with ethyl cellulose as an organic binder and α-terpineol as a liquid phase. The resulting viscous pastes were further homogenized in an ultrasonic bath for 30 min at 30 °C to achieve stable, finely dispersed colloidal pastes. Lastly, these pastes of S1, ATO1, and ATO2 were drop-coated on the microheater devices, as described in Section 2.3. By drop-coating method, the pastes were spread on the microheater devices. The average thickness of the deposited films was within 30–45 um. Next, the gas sensing devices were treated at 220 °C for 30 min to remove the organic media, and then at 650 °C for 2 h to stabilize the active material on the substrate. Finally, the devices were bonded on standard TO39 support by ball bonding using gold wires (see inset in Figure 1), and placed on our customized sensing measurement system for electrical characterizations [54,55].

### 2.4. Gas Sensing Measurements

The sensing film resistance was measured in a sealed gas test chamber, which is linked to a peripheral pneumatic system composed of mass flow controllers and gas cylinders with certified concentrations (Figure 1). The input voltage of the microheater was adjusted to obtain the proper working temperature for the sensing materials, knowing the temperature coefficient of resistance (TCR) of the heater. Film resistance was continuously measured by the data acquisition [44]. Synthetic dry air with a total flow rate of 200 sccm was injected into the test chamber for 2 h in order to achieve a controlled steady-state dried atmosphere. The microheater input voltage was set to obtain an operating temperature of 200 °C, 300 °C, and 400 °C, until the surface of the sensing materials reached a thermodynamically stable situation. Tested gases were mixed with dry air in order to obtain the expected analyte concentration values. The target gas injection, after the stabilization of the baseline sensor signal, was set to 30 min. To deeply investigate the selectivity of the sensing materials, different gases in dry air were tested in this work: acetaldehyde (2 ppm), ethanol (5 ppm), acetone (5 ppm), H_2_S (5 ppm), CO (20 ppm), ethylene (50 ppm), CO_2_ (400 ppm), NH_3_ (100 ppm), and NO_2_ (10 ppm). Gas concentrations were chosen based on threshold limit values (TLV). Then, sensing characterizations were carried out at different relative humidity percentages (RH%), by exploiting a bubbler system filled with deionized (DI) water and being connected to the pneumatic system. To investigate the humidity influence on the gas sensing performance, temperature and relative humidity in the gas chamber were monitored by a commercial HIH-4000 Honeywell sensor (accuracy = ±3.5%) [45,46].

To analyze the dynamical response vs. concentration under different gases, the response value was defined by the following equations:

R = (R_air_ − R_gas_)/R_gas_ (in reducing gases) (1)

R = (R_gas_ − R_air_)/R_air_ (in oxidizing gases) (2)

In which R_gas_ and R_air_ are the resistance values measured under target gases and air, respectively.

## 3. Results

### 3.1. Morphological and Elemental Studies

**Figure 2:**
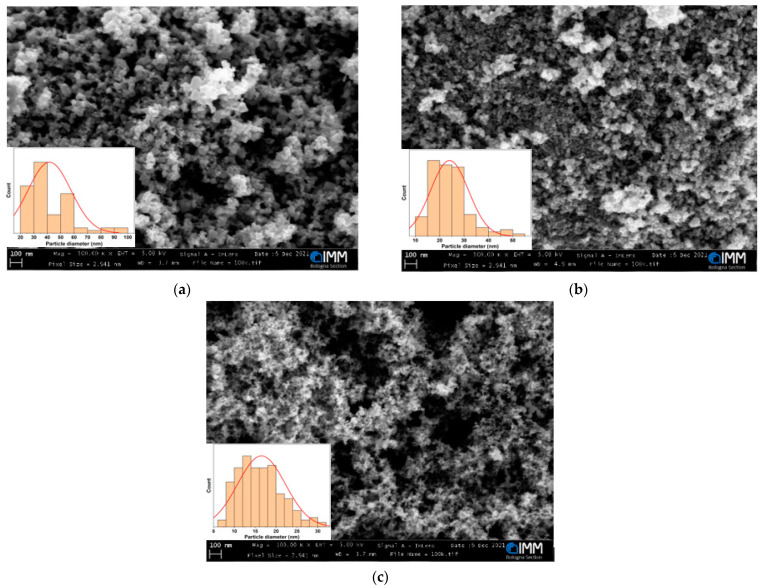
SEM images of (**a**) S1; (**b**) ATO1 and (**c**) ATO2 at 250 kX magnification. Insets are the histograms of grain size distribution for sample S1 (**a**), ATO1 (**b**) and ATO2 (**c**).

The characterizations were performed onto the commercial powders as received, without any further treatment. The surface morphology of the samples was investigated by using SEM, and the results are shown in Figure 2, where all the sample powders show a spherical-like shape. Figure 2a represents the nanostructure of pure SnO_2_, whose particles size mostly is bigger than 30 nm, and the average size calculated from ImageJ software is 41.65 ± 15.97 nm, which are shown in Figure 2a inset. ATO1 exhibits big particle clusters among the nanopowders (in Figure 2b), and the particle size distribution shows a bell-shaped curve with average size of 24.04 ± 7.79 nm as seen in the inset figure of Figure 2b. It is very obvious in Figure 2c that the size of ATO2 is the smallest, and the distribution is homogeneous with an average size of 16.49 ± 5.71 nm seen from Figure 2c inset. It is well worth noting that increasing the antimony doping level in SnO_2_ usually leads to a decreased particle diameter, which suggests that antimony doping inhibits the coalescence of the SnO_2_-based particles during the powder calcination. Growth of the nanoparticles during high temperature thermal treatment can be explained by referring to the surface energy [56,57,58]. Indeed, smaller particle sizes result in high surface energy, which can be conveniently minimized, during the heat treatment by grain coalescence, which naturally results in a reduction of the surface area with a concomitant increase in particle size. Thus, the presence of antimony doping leads to a decline of the surface energy of SnO_2_ compared to pure SnO_2_, which results in small particle size formation during the nanoparticle synthesis and also hinders nanoparticle coalescence during the heat treatment [56,58].

**Figure 3:**
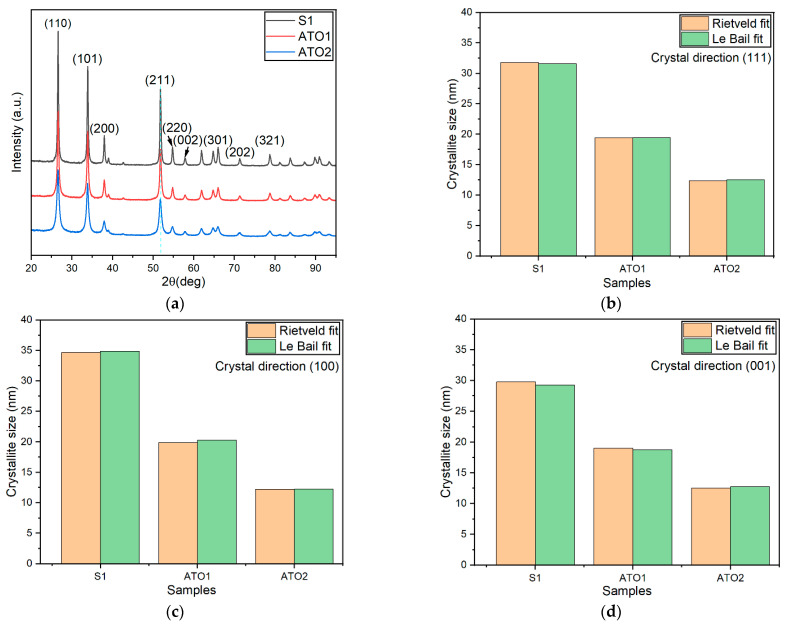
X-ray diffraction patterns (**a**) and crystallinity size (**b**–**d**) of S1, ATO1, and ATO2 estimated by the Rietveld and Le Bail approach from XRD data analysis, in the three directions of (111), (100), and (001).

The crystal structure of S1, ATO1, and ATO2 were evaluated by XRD analysis. Figure 3a presents the XRD patterns of the powders at room temperature. As can be seen in this figure, all the XRD diffraction peaks can be easily indexed to tetragonal crystallized rutile structure of SnO_2_, which corresponds to PDF 00-041-1445, P42/nmn. The typical peaks of rutile crystal phase are identified, i.e., (110), (101), (200), and (211), located at 26.6°, 33.89°, 37.95°, and 51.78°, respectively. Table 1 displays the lattice parameters a and c estimated from XRD analysis; it can be seen that the parameters a and c increased slightly after doping, due to the larger radius of Sb ions replacing Sn ions in the lattice [39]. However, neither Sb_2_O_3_ with a cubic structure (PDF 00-043-1071, 00-005-0534) or an orthorhombic structure (PDF 00-011-0689), nor Sb_2_O_5_ with a monoclinic structure (PDF 00-033-0110) peaked were found in Figure 3a. This result demonstrates the success of the doping process, because Sb ions take the place of Sn ions in the crystal structure, without forming any additional and undesired Sb*_y_*O*_x_* phases [59]. In addition, it is important to find out the effects of antimony doping on SnO_2_ material from Figure 3a, where the width of the peaks increased after antimony doping level enhancing. Since wider full-width peaks indicate the decrease in crystallite size, Sb doping can suppress the crystallite growth of SnO_2_. Figure 3b–d show the crystallite size of samples in different crystal directions (111), (100), and (001), respectively, which proves that an enlarged doping level resulted in a small crystallite size [60]. The reason for this result can be ascribed to the successful substitution of Sn ions with Sb into SnO_2_ lattice [37,61].

The qualitative and quantitative analyses of these materials elements were performed by XRF and EDS techniques. Since oxygen cannot be detected by exploiting XRF, only Sn and Sb elements are compared in different samples. S1 contains 99.87% of Sn element and 0.13% of impurities (mainly Al). ATO1 includes 89.89 at% Sn and 10.12 at% Sb, and the ratio of Sb/Sn is 11.26%. ATO2 contains 85.19 at% Sn and 14.81 at% Sb, and the ratio of Sb/Sn is 17.38%, which is shown in Table 2.

Table 3 shows the elemental composition in the obtained samples using EDS analysis. The atoms ratio of Sn/O for S1 is 41.66%, which means the material S1 is not perfectly stoichiometric as a molecular formula of SnO_2_. The atoms ratios of Sn/O in ATO1 and ATO2 are 50.09% and 45.57%, which are both higher than that of pure SnO_2_. A lower O atom concentration in SnO_2_ could indicate higher O vacancies; hence, Sb doping increased the O vacancies in ATO1 and ATO2 compared to the higher O atoms content in S1. The atom ratios of Sb/Sn in ATO1 and ATO2 are 9.25% and 15.02%, which are lower than the results obtained from XRF.

**Figure 4:**
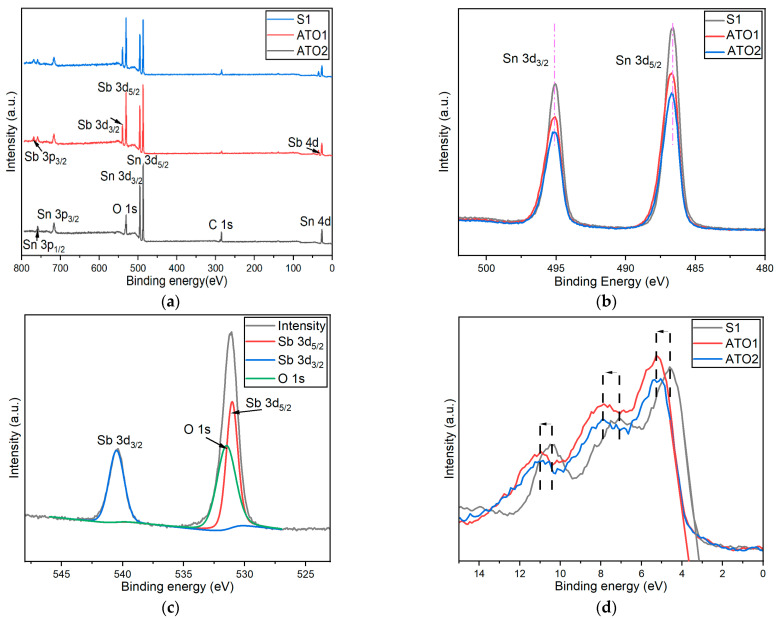
XPS spectra of S1 and ATO1 and ATO2: (**a**) survey spectrum, (**b**) Sn 3d spectrum, and (**c**) Sb 3d spectrum of ATO2. (**d**) XPS valence band of the three samples.

The surface elemental compositions and chemical states of the prepared samples were further investigated by XPS. A wide scan spectrum of pure and doped SnO_2_ samples is shown in Figure 4a, with peak assignment. Both Sb and Sn peaks are present, along with carbon and oxygen peaks. The binding energy of spin orbital states Sn 3d_5/2_ and Sn 3d_3/2_ are 486.5 eV and 495.0 eV, respectively, and the peak separation is 8.5 eV, confirming the existence of Sn^4+^ oxidation state in samples, which can be found in Figure 4b [60]. The doping element plays an important role in the chemical interaction, particularly in changing of the covalency between O 2p and cation Sn 5p states. In our samples, the introduction of Sb in SnO_2_ results in a small binding energy shift of Sn 3d peaks compared to pristine SnO_2_ (Figure 4b). Due to a partial overlap between Sb 3d_5/2_ and O 1s, it is necessary to fit the peaks in the O 1s/Sb 3d region in order to quantify both oxygen and antimony [34,62]. The Sb 3d_5/2_ and Sb 3d_3/2_ spin-orbital spectra are assigned to binding energies 530.3 eV and 540.2 eV. In order to determine the relative concentration of Sb^3+^ and Sb^5+^ in Sb/SnO_2_ samples, accurate deconvolution of Sb 3d peaks is required. However, the overlap with the O1s core level makes it rather complicated and unreliable. Nevertheless, the binding energy of Sb 3d_3/2_ suggests the coexistence of the Sb^3+^ and Sb^5+^ oxidation states, which has been constantly observed in ATO materials (Figure 4c) [40,62,63].

For the XPS investigation of these samples, it is critical to determine the relative concentrations of Sn and Sb on the surface of nanoparticles. In our samples, the obtained atom ratios between elements Sb and Sn are 42.93% and 50.79% for ATO1 and ATO2, respectively. This result is very different from the data obtained by XRF and EDS. Since electrons from 10 nm or less deep on the surface of samples will contribute the most XPS spectra, the information received from XPS is extremely surface sensitive. XRF and EDS techniques are able to analyze approximately a thickness of more than 1 um. Comparing these two results, one can observe that the concentration of antimony on the surface is much higher than that inside the crystal. It appears that a segregation phenomenon due to high doping levels occurs, which could be caused by different ion geometric size or reactivity difference between Sn ions and Sb ions. This behavior has been already observed for other highly doped nanomaterials [64,65]. The segregation can occur either during the synthesis of the nanoparticles or during the calcination of the powder. The surface segregation of a dopant is intrinsically connected to a reduction of the surface energy of the host materials, as described by the Gibbs adsorption isotherm equation for two components [65].

The valence band (VB) obtained from XPS gives information about the density and occupancy of electronic states in the valence band of the materials. VB photoelectron spectra for the three samples are presented in Figure 4d. From this figure, it can be seen that the spectra display the characteristic three-peaked structure of SnO_2_ coming from O2p states. The onset of the valence band, as defined by linear extrapolation of the valence band edge, reveals a shift towards 3.697 eV upon doping with Sb, whereas it was at 3.140 eV for the undoped sample. The shift of 0.557 eV is ascribed to the occupation of conduction band states in degenerately doped SnO_2_. Shifts of the three characteristic valence band peaks are also found, and are reported in Figure 4d. In addition, the recorded shifts indicate the stabilizing contributions to the valence band by the formation of Sb-O bonds. This explains the energy gained by replacing Sn ions with Sb species at the surface, the lower surface energy discussed before, and the enhanced surface concentration of Sb(V) species [62,63].

**Figure 5:**
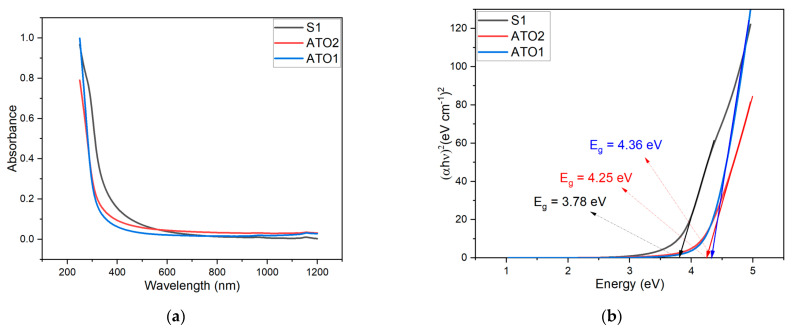
(**a**) UV-visible spectra of the S1, ATO1, and ATO2 powders; (**b**) Tauc plots obtained by the UV-visible spectra for the direct band-gap calculation.

The UV-visible analysis was carried out to determine the optical band gap of the three samples. From the absorption spectra of the films (Figure 5a), the band gap can be determined using the Tauc plot method. The optical band gap of pure SnO_2_ film (3.78 eV) is the smallest among the three samples (as shown in Figure 5b). With the addition of Sb, the optical band gap of the films increased to 4.36 eV for ATO1. As the amount of Sb doping rises, the optical band gap for ATO2 lowers slightly to 4.25 eV. The band gap of ATO powders is greater than that of pure SnO_2_ film and it decreases as Sb doping increases. The Burstein-Moss effect [66] explains this phenomenon: as doping in a semiconductor increases, the band gap of the film changes, and the unoccupied energy levels in the valence band top and the conduction band separate due to the Pauli exclusion principle. The Fermi energy level penetrates the conduction band in n-type doped semiconductors. When some electrons are filled in the conduction band due to the high carrier concentration, the electrons require more energy for the transition from the valence band to the conduction band, causing the band gap of the film to widen.

The ionized impurity formed by the high concentration doping ions will attract the carriers and cause the conduction band to migrate down when the Sb doping quantity is too high. At the same time, the high doping concentration will increase the number of defects in the film, the wave functions of the defect state will overlap [67], and the film band gap will narrow. Furthermore, a quantum size effect contribution could be considered especially for ATO samples, which involves a shift to higher energy of the band gap of nanopowders compared to the corresponding bulk materials

### 3.2. Gas Sensing Characterization

When the resistance values of functional materials change, the concentration of the free carriers on the surface and in the bulk also changes based on Morrison’s equation, which can reflect the height of the surface potential barrier changing [40,68]. The resistances of the sensing materials were measured by using our customized system (seen in Section 2.4) at different operating temperatures (200, 300, and 400 °C). The baseline resistance values in dry air of these three produced devices are shown in Table 4, where it is clarified that doped SnO_2_ possesses a much lower resistance than pure SnO_2_. It is because antimony doping inserts a new energy level (donor level) within the energy band gap near the conduction band of SnO_2_. The donor level in SnO_2_ makes it easy for electrons with sufficient energy to jump to the conduction band, contributing to the conductance mechanism [36,69]. Furthermore, with the temperature rising, the resistances of S1 and ATO1 fall, while ATO2 resistances increase. However, by comparing ATO1 and ATO2, it is clear that these two samples present opposite resistance changing trends with increasing temperature. This behavior was previously reported in [37]. The reason for this is that high doping concentrations cause a significant increase in the number of trap states due to excessive lattice defects [70], which dominate the carrier concentration above the critical level of Sb, lowering the film’s conductivity. Furthermore, the scattering effect [71] of high dopant ion concentrations on carriers would reduce carrier mobility in the film, lowering the film’s conductivity.

**Figure 6:**
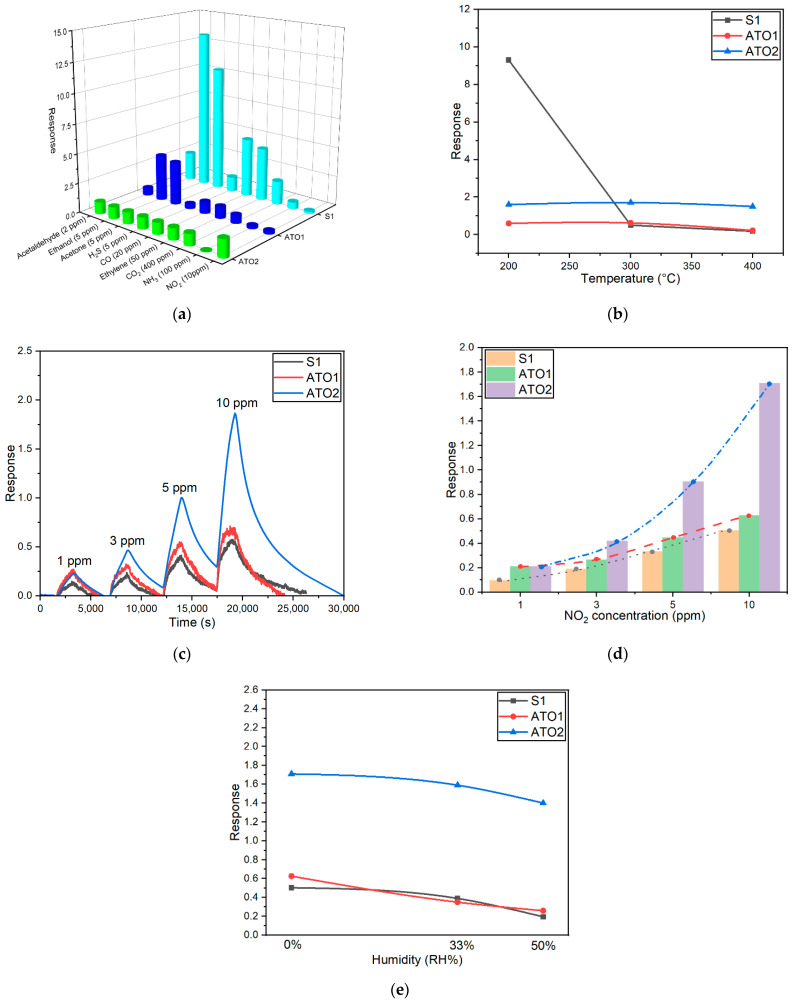
Electrical characterizations of the fabricated sensors: (**a**) response to different gases at 400 °C in dry air; (**b**) response to 10 ppm of NO_2_ at different operating temperatures in dry air; (**c**) dynamical response of the three sensors towards 1, 3, 5, and 10 ppm NO_2_ at 300 °C; (**d**) variation of the response vs. NO_2_ concentrations for the three sensors in dry air at 300 °C; (**e**) response to different humidity at 300 °C under 10 ppm NO_2_.

Selectivity of the produced devices was tested towards various target gases according to the TLV of each analyte (2 ppm of acetaldehyde, 5 ppm of ethanol, 5 ppm of acetone, 5 ppm of H_2_S, 20 ppm of CO, 50 ppm of ethylene, 400 ppm of CO_2_, 100 ppm of NH_3_, and 10 ppm of NO_2_) at 400 °C as operating temperature. The results in Figure 6a highlight an enhancing of the response towards NO_2_ of the ATO2 with respect to pristine SnO_2_, demonstrating, in this way, the pivotal role of Sb doping in the sensing mechanism of the material towards NO_2_.

After the assessments of selectivity properties of the sensing films, the sensors were tested vs. NO_2_ at different working temperatures, ranging from 200 to 400 °C. Responses in dry air are shown in Figure 6b, highlighting that the highest sensing response for ATO1 and ATO2 was obtained at 300 °C. Furthermore, a pure SnO_2_ response showed a very strong drop by changing the operating temperature from 200 to 300 °C, while the ATO1 and ATO2 responses fell slightly. The opposite trend of ATO and SnO_2_ sensors led to a higher sensing response of ATO2 than SnO_2_ at operating temperatures of 300 and 400 °C.

In order to study the sensitivity of the functional materials towards NO_2_, the sensors were exposed to different concentrations (1, 3, 5, and 10 ppm) of NO_2_ at the best working temperature for ATO1 and ATO2 (i.e., 300 °C). Figure 6c reveals that raising up the NO_2_ concentrations, the responses of S1 and ATO1 increased almost linearly with the increase of NO_2_ concentrations, while ATO2 possesses an exponential increase in the sensor response, which indicates that ATO2 is more sensitive to NO_2_ than S1 and ATO1, and the trend is shown in Figure 6d.

The influence of relative humidity on the sensing response and on the materials’ resistance was studied and reported in Figure 6e and Table 5. The sensors were exposed to 10 ppm of NO_2_ in the presence of 33 and 50 RH% humidity at 300 °C, to observe the behavior of the devices in real-life environments compared to dry air. Figure 6e highlights that the presence of moisture in the gas chamber slightly decreased the sensing response of the three sensors to NO_2_. Furthermore, ATO2 showed around three times larger response than pristine SnO_2_ and ATO1 both in dry and wet air. Although the drop of the sensing response to NO_2_ in wet air for all sensing devices is mild, it hints at the role of water molecules in the surface reaction mechanism. When the humidity increases, all samples exhibit decreasing response signals and decreasing resistance values, which suggests that the humidity declines the sensing materials performance. However, one can observe that especially for ATO2 the film resistance is not affected by humidity increase since the values obtained at 33% and 50% RH are almost the same (Table 5).

## 4. Gas Sensing Mechanism Discussion

The suggested model demonstrates that the gas sensing mechanism of metal-oxide semiconductors can be summarized as adsorption-oxidation-desorption processes. At an operating temperature above 100 °C, the adsorbed oxygen molecules on the surface of the sensing material will be ionized by capturing electrons from the conduction band of the materials [1].

O_2_ (air)→O_2_ (adsorbed)→O_2_^−^ (low temperature) or O_2_^−^ (high temperature) (3)

In the SnO_2_ case, a depletion layer will be formed on the surface after oxygen adsorption, and when it is exposed to the gas target (NO_2_ in this case), NO_2_ will take electrons from the conduction band of sensing material (Equation (4)):

NO_2_(gas) → e^−^ + NO_2_^−^(adsorbed) (4)

**Figure 7:**
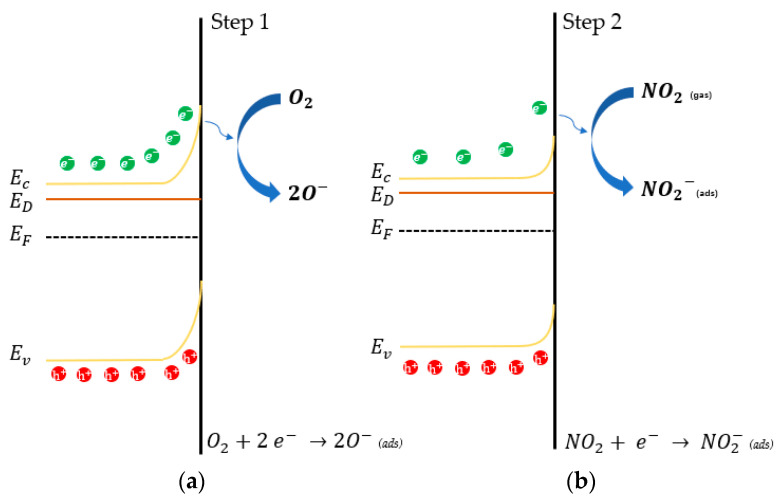
Energy band diagrams illustrating the sensing mechanism of the samples toward NO_2_. (**a**) adsorption of oxygen species from the surrounding atmosphere and (**b**) the interaction with the target gas (i.e., NO_2_).

The sensing mechanism of the sensors can be divided into two main steps: (i) the adsorption of the oxygen species from the surrounding atmosphere when the gas sensor device is exposed to the air and (ii) the interaction with the analyte. In the first step (Figure 7a), during the adsorption of oxygen from the surrounding atmosphere, the electrons accumulate at the surface of the sensing material, creating a depletion zone (band bending effect), affecting the electron affinity, and achieving an equilibrium translated to the sensor baseline [5]. In the second step (Figure 7b), during the interaction with the target gas (i.e., NO_2_), the accumulated electrons participate in this reaction, resulting in a decrease of the potential barrier and increasing the conductivity of the material that is translated to the gas sensor response. Sb doping created a new energy band near the conduction band of SnO_2_ (i.e., donor level). As a result, there is a greater concentration of free charge carriers, and the Fermi level moves closer to the conduction band [40]. Increasing the concentration of Sb leads to a decrease in the band gap value, which can be explained by the movement of the conduction band minimum as well as the fermi level [72]. The closer the donor level is to the conduction band minimum as well as the higher the concentration of the donors is, the greater the available sites for the adsorption of oxygen species on the surface of the material and the better the sensor response.

During the whole sensing process, the resistance of sensing material will change depending on its own physical properties and on the gas target. After Sb doping, a new donor level inside the conduction band of SnO_2_ is brought about, which is beneficial to electron transfer and results in lower resistance of sensing materials. The higher content of antimony results in a more conductive film in air with additional carrier availability. In addition, Sb doping could also produce crystal defects, which affects oxygen vacancy quantity.

At operating temperatures of 300 °C and 400 °C, ATO samples showed an amplified response under NO_2_ exposure with respect to pure SnO_2_, which indicates that higher Sb doping causes an enhanced response. Considering the high electron affinity of NO_2_, and its oxidizing behavior, there are two ways that probably explain this phenomenon: (i) the decrease of the band gap of ATO and (ii) the increase of oxygen vacancies in ATO samples, caused by Sb doping. In particular, oxygen vacancies can optimize the chemisorption of oxidizing gases from ambient air, and provide more surface adsorption sites. In addition, ATO powders showed a smaller particle size, which suggests a bigger active surface area compared to pristine SnO_2_, providing, in this way, more adsorption sites available for reaction with the analyte gas. However, they did not show any enhanced response in these above-mentioned gases except for NO_2_. The response not only depends on the geometry parameters, but is also affected by the surface chemical reactions, which is related to the donor-acceptor level of materials, and induces boundary barrier changing. It is apparently clear that in our case, the surface chemical reaction prevails during the whole sensing process. In addition, the decrease of the sensing response to NO_2_ of both SnO_2_ and ATO sensors at 400 °C could be due to the collapse of defective sites at high temperatures [28].

When the atmosphere changed into a near-real environment with a humidity of 33% and 50% of RH%, all samples showed a fading response trend and a decreased resistance value. In this case, water molecules can occupy reactive sites on the surface of nanoparticles, involving a decrease in the gas sensing response by injecting NO_2_ in the gas chamber in the presence of moisture [73]. Additionally, water molecules can provide H^+^ and OH^−^ ions, influencing the conductivity of the sensing materials.

## 5. Conclusions

In our study, the sensing performance of highly doped Sb/SnO_2_ nanoparticles were studied and compared to pristine SnO_2_. SEM analyses demonstrated that antimony doping affects the morphology of SnO_2_ powder, by suppressing the nanoparticles growth. In addition, antimony doping also hindered the crystallite size and expanded lattice parameters due to larger Sb^3+^ ions integration into SnO_2_ as shown in XRD analyses. XRF and EDS investigations approved the Sb/Sn atoms ratio of ATO1 and ATO2 and revealed the lower O atom content after samples doping, which could enhance the O vacancy concentration and the conductivity. The comparison of XPS analysis to XRF and EDS results in a different concentration of antimony highlighting its possible segregation on the nanoparticles’ surface. The electrical measurements showed that ATO sensors possessed much lower resistance due to the introduction of Sb in the SnO_2_ lattice, which results in the addition of a new energy band close to the conduction band, and then in an increased conductivity of the sensing film. The investigation of the sensing performance demonstrated a good selectivity of ATO sensors to NO_2_ among the gases analyzed at 400 °C in dry air. Furthermore, the sensing response of ATO1 and ATO2 were just slightly affected by humidity. In particular, ATO2 showed an insensitivity increasing the humidity concentration from 33 to 50%RH, making them promising sensitive materials for the detection of NO_2_ in the real atmosphere.

## Figures and Tables

**Table 1:** Lattice parameters of S1, ATO1, and ATO2 samples estimated by Rietveld and Le Bail approach from XRD data analysis.

Samples	Lattice Parameter *a* (Å)		Lattice Parameter *c* (Å)	
	Rietveld Fit	Le Bail Fit	Rietveld Fit	Le Bail Fit
S1	4.738377 (60)	4.738349 (60)	3.186908 (57)	3.186859 (56)
ATO1	4.73853 (10)	4.73862 (10)	3.188074 (93)	3.188113 (94)
ATO2	4.73991 (14)	4.74024 (15)	3.18913 (12)	3.18917 (13)

**Table 2:** Elemental composition of samples obtained from XRF analysis.

Samples	Sn (at%)	Sb (at%)	Sb/Sn (%)
S1	99.87 ± 0.64	-	-
ATO1	89.88 ± 0.67	10.12 ± 0.09	11.26
ATO2	85.19 ± 1.68	14.81 ± 0.11	17.38

**Table 3:** Elemental composition of samples obtained from the EDS analysis.

Samples	Sn (at%)	Sb (at%)	O (at%)	Sb/Sn (%)
S1	29.41 ± 1.76	-	70.59 ± 4.31	-
ATO1	32.34 ± 1.96	2.99 ± 0.38	64.67 ± 3.24	9.25
ATO2	29.90 ± 1.90	4.49 ± 0.44	65.61 ± 3.47	15.20

**Table 4:** Resistance (kΩ) of samples at different working temperatures in dry air.

Sample\Temperature	200 °C	300 °C	400 °C
S1	882.3	251.6	156.6
ATO1	242.2	106.2	78.2
ATO2	33.9	85	91.3

**Table 5:** Resistance (kΩ) of samples at different humidity at 300 °C.

Sample\Temperature	0 RH%	33 RH%	50 RH%
S1	378.15	106.20	78.23
ATO1	173.11	56.17	46.784
ATO2	229.50	39.54	41.51

## Data Availability

The data that support the findings of this study are available from the corresponding authors upon request.
